# The Prevention Research Centers Healthy Aging Research Network

**Published:** 2005-12-15

**Authors:** Jason E Lang, The Healthy Aging Research Network Writing Group, Lynda Anderson, LoGerfo James, Joseph Sharkey, Elaine Belansky, Lucinda Bryant, Tom Prohaska, Mary Altpeter, Victor Marshall, William Satariano, Susan Ivey, Constance Bayles, Delores Pluto, Sara Wilcox, R. Turner Goins

**Affiliations:** Health Care and Aging Studies Branch, Division of Adult and Community Health, National Center for Chronic Disease Prevention and Health Promotion, Centers for Disease Control and Prevention; Mr Lang is a member of the Healthy Aging Research Network Writing Group; Division of Adult and Community Health, Centers for Disease Control and Prevention, Atlanta, Ga; Health Promotion Research Center, University of Washington, Seattle, Wash; Texas Healthy Aging Research Network Center, Texas A&M University, College Station, Tex; Rocky Mountain Prevention Research Center, University of Colorado, Denver, Colo; Rocky Mountain Prevention Research Center, University of Colorado, Denver, Colo; Center for Research on Health and Aging, University of Illinois at Chicago, Chicago, Ill; Institute on Aging, University of North Carolina at Chapel Hill, Chapel Hill, NC; Institute on Aging, University of North Carolina at Chapel Hill, Chapel Hill, NC; University of California, Berkeley, Berkeley, Calif; University of California, Berkeley, Berkeley, Calif; Center for Healthy Aging, University of Pittsburgh, Pittsburgh, Pa; Rocky Mountain Prevention Research Center, University of Colorado, Denver, Colo; Prevention Research Center, University of South Carolina, Columbia, SC; Robert C. Byrd Health Sciences Center School of Medicine and Center on Aging, West Virginia University, Morgantown, WVa

## Abstract

**Background:**

The Prevention Research Centers Healthy Aging Research Network (PRC–HAN), funded by the Centers for Disease Control and Prevention's (CDC's) Healthy Aging program, was created in 2001 to help develop partnerships and create a research agenda that promotes healthy aging. The nine universities that participate in the network use their expertise in aging research to collaborate with their communities and other partners to develop and implement health promotion interventions for older adults at the individual, organizational, environmental, and policy levels.

**Context:**

The population of older adults in the United States is growing rapidly; approximately 20% of Americans will be aged 65 years or older by 2030. The health and economic impact of an aging society compel the CDC and the public health community to place increased emphasis on preventing unnecessary disease, disability, and injury among older Americans.

**Methods:**

The PRC–HAN has a broad research agenda that addresses health-promoting skills and behaviors, disease and syndrome topics, and knowledge domains. The network chose physical activity for older adults as its initial focus for research and has initiated two networkwide projects: a comprehensive, multisite survey that collected information on the capacity, content, and accessibility of physical activity programs for older adults and a peer-reviewed publication that describes the role of public health in promoting physical activity among older adults. In addition to participating in the core research area, each network member works independently with its community committee on PRC–HAN activities.

**Consequences:**

As a result, the network is 1) expanding prevention research for older adults and their communities; 2) promoting the translation and dissemination of findings to key stakeholders; 3) strengthening PRC–HAN capacity through partnerships and expanded funding; and 4) stimulating the adoption of policies and programs by engaging policymakers, planners, and practitioners. In 2003, the PRC–HAN initiated an internal evaluation to better define the network's contributions to healthy aging, formalize internal processes, and better equip itself to serve as a model for other PRC thematic networks. The PRC–HAN is conducting a pilot evaluation for eventual inclusion in the PRC national evaluation.

**Interpretation:**

The PRC–HAN has established itself as an effective research network to promote healthy aging. It has developed trust and mutual respect among participants, forged strong ties to local communities, and shown the ability to combine its expertise in healthy aging with that of partners in national, state, and local organizations.

## Background

In 2001, the Centers for Disease Control and Prevention's (CDC's) Healthy Aging program issued a request for proposals to create a Prevention Research Centers Healthy Aging Research Network (PRC–HAN). The plan for this network was guided by the principles underlying academic–community partnerships and coalitions (1,2) and had several goals. First, the network would foster a strong sense of local program ownership by drawing on the Prevention Research Centers' (PRCs') established community relationships to collaboratively develop a healthy aging research agenda. Second, facilitating partnerships among PRCs would strengthen the focus, cohesiveness, and collaboration of research on aging. Third, by combining the diverse perspectives of network members and establishing new national linkages, the network would have the potential to develop and promote interventions at individual, organizational, environmental, and policy levels. Fourth and finally, the network would link the CDC's Healthy Aging program to additional prevention researchers outside of the CDC. Here we describe the evolution of the PRC–HAN's structure, mission, and research agenda. We then provide several examples of network activities that are helping the CDC and its partners to meet the challenges of an aging U.S. population. We conclude with planned evaluation activities and next steps.

## Context

### Changing demographics in the United States

Both the number and the percentage of older adults (aged 65 or older) in this country are increasing at a much faster rate than ever before. This population will continue to grow, doubling from 35 million in 2005 to 70 million by 2030, when one in every five Americans will be aged 65 years or older (3). The looming health and economic impact of an aging society compels the CDC and the public health community to increase emphasis on preventing unnecessary disease, disability, and injury among older Americans. An enhanced focus on prevention is critical to preserving older individuals' independence and reducing long-term care needs. This enhanced focus on prevention is also critical for helping to stem escalating health care costs. Fortunately, our increased understanding of the role of risk factors such as physical inactivity in the onset and progression of chronic illness and disability has made this goal achievable.

### The CDC's Prevention Research Centers (PRC) Program

The PRC Program, which includes 33 academic centers in 26 states, was established in 1984. (More information on the program is available from www.cdc.gov/prc)The PRC Program, which includes 33 academic centers in 26 states, was established in 1984. (More information on the program is available from www.cdc.gov/prc). The centers are selected through a competitive peer-review process. All funded PRCs share a set of common principles, including 1) a focus on community-based participatory research that contributes to the knowledge base and informs public health programs and policies; 2) the creation of partnerships among communities, health departments, and other groups; 3) the timely collection, synthesis, and dissemination of the results of research and programs; 4) education and training in prevention research for the community, policymakers, health advocacy groups, and others; and 5) strong collaborative ties to the CDC PRC Program office for ongoing network enhancement and evaluation (4). The research focus of each PRC reflects the scientific expertise of its faculty and the goals of its community partners. Each PRC establishes a committee of community leaders and organizations to build a long-term relationship for engaging communities as partners in research and dissemination efforts. In addition to conducting a core research project within the area of research focus, the centers work with partners and are eligible to apply for Special Interest Projects (SIPs) Competitive Supplements, which are defined by the CDC and other federal agencies.

## Methods

### Creation of the PRC–HAN

Through the PRC SIPs competitive process in 2001, the University of Washington; the University of California, Berkeley; the University of Colorado; the University of Illinois at Chicago; the University of North Carolina at Chapel Hill; and the University of South Carolina became the first members of the PRC–HAN. The University of Pittsburgh was added to the network in 2002, and Texas A&M University and West Virginia University joined in 2004. All of the centers funded in 2001 competed and were refunded in 2004. For more information on the PRC–HAN, see http://depts.washington.edu/harn/.

### Structure and communications

The participants from each university bring their unique perspectives and those of their communities to the network. Two of the network's PRCs have healthy aging as a focus of their core programs. The other network members represent various aging centers and programs from the broader universities that house their core PRC programs. Each member of the PRC–HAN has an equal voice in a consensus-based decision-making process that includes the agreement of all funded centers and key partners, such as the CDC, when selecting research topics and identifying the number of network centers that will participate in a given activity. The network structure allows additional research expertise and community capacity to be brought in from outside the network membership to help investigate new topics. The University of Washington Health Promotion Research Center (UWHPRC) is designated as the coordinating center. UWHPRC receives additional funds to support coordinating activities, which include initiating regular network communications, acting as a clearinghouse for information among network members, and serving as the PRC–HAN's primary representative to outside organizations and groups.

The CDC's Healthy Aging program funds the infrastructure of the PRC–HAN. During the PRC–HAN's first year, its members collaborated with the CDC to formulate a common mission, establish a definition of healthy aging, and develop a framework and agenda for research in public health and aging. The CDC SIPs and external partners continue to support the work of the network.

### Mission

The mission of the PRC–HAN is to better understand the determinants of healthy aging in older adult populations, to identify interventions that promote healthy aging, and to help translate this knowledge into sustainable community-based programs throughout the nation. The PRC–HAN defines healthy aging as the development and maintenance of optimal physical, mental, and social well-being and function in older adults. It is most easily achieved when physical environments and communities are safe and support the adoption and maintenance of attitudes and behaviors known to promote health and well-being and when health services and community programs are used effectively to prevent or minimize the impact of acute and chronic disease on function. This definition reinforces a social–ecological view of the determinants of health (5). The PRC–HAN adapted and applied a social–ecological framework first developed by McLeroy et al and later refined by Sallis et al (5,6). This model emphasizes the interactions among individual, group, and community levels while also considering the environmental and policy arenas. The model is available from http://depts.washington.edu/harn/research%20agenda/research%20agenda.shtml.

The research agenda developed by the PRC–HAN is intentionally broad. The agenda is built upon the intersections of three areas: disease topics, health-promoting skills, and knowledge domains (Table). The diseases and conditions that the agenda addresses are leading causes of illness and death among older adults. The second intersecting area of the agenda consists of six key health-promoting skills and behaviors. Although many of the skills and individual behaviors are analogous to the concept of underlying causes of illness and death, the agenda has expanded the usual list to include skill building for self-management and involvement in social activity (7). Finally, the agenda points to five primary areas of knowledge required to translate topics from research to policy arenas. These five domains allow the PRC–HAN to determine priority areas for the network by identifying risk factors, their impact on health and well-being, and behavioral, social, and environmental strategies to reduce them. 

### Objectives

Grounded in the philosophy of participatory research and collaboration, the network strives to 1) expand prevention research for older adults and their communities; 2) promote the use of research findings by translating and disseminating them to stakeholders; 3) strengthen PRC–HAN capacity through partnerships and expanded funding; and 4) stimulate the adoption of policies and programs by engaging policymakers, planners, and practitioners in the development process. Consistent with the national PRC Program, each network member works with its own community committee on PRC–HAN activities.

### Research activities

Through a consensus process, the network chose physical activity for older adults as its initial focus for research. As an example of the ecological framework within which the PRC–HAN works, one research question of particular interest was how barriers within the built environment limit the amount of physical activity that older adults engage in.

Two PRC–HAN projects have involved all network members. The first of these, led by the University of Illinois at Chicago and modeled after the *Get in Shape Chicago* guide (available from http://shapechicago.com), was a comprehensive, multisite survey that collected information on the capacity, content, and accessibility of physical activity programs for older adults. The workgroup developed a common survey instrument, refined the survey and analysis methods, tracked field activities, and coordinated data collection and analysis in the PRC–HAN community sites. The PRC–HAN members used the results of this survey to develop local resource guides for their communities. The project will track community distribution and use of the guides, which will soon be available online. The survey results are also being used to develop a publicly available interactive Web guide that can be applied in any community. An example of a draft guide is available from www.shapeupkingcounty.org.

The second networkwide project was the development of a peer-reviewed publication, which is currently under review. This publication describes the role of public health in promoting physical activity among older adults. The in-depth, evidence-based review has become a template for how the PRC–HAN will prepare for new topic areas. Further, the messages emerging from this effort will form the basis for training and educational materials that will be shared with constituents in the public health and aging services network.

The PRC–HAN conducts many of its activities through workgroups. The environmental factors workgroup is addressing issues associated with the physical environment and its relationship to physical activity among older adults. The University of California, Berkeley serves as lead center for this workgroup. Reaching beyond the expertise of the current PRC–HAN members, the workgroup collaborated with researchers at St Louis University PRC to develop and refine an instrument for assessing environmental factors that affect the walkability of neighborhoods for older adults. Using a revised instrument based on their previous work, St Louis University researchers trained PRC–HAN members on how to conduct audits. These PRC–HAN members then conducted pilot environmental audits in each of their communities. These data are currently being analyzed and will be used to refine the final instrument, along with qualitative data from interviews conducted with older adults to determine their reasons for walking or not walking. This workgroup has developed a directory of Web sites related to environmental assessment and a manuscript assessing the current knowledge base and next steps for research. Finally, members of the network have received funding from The Robert Wood Johnson Foundation Active Living by Design program to assess the environmental correlates of physical activity among older adults. The project will include environmental assessments and interviews with older adults in five areas covered by PRC–HAN member sites.

Additional workgroups have been formed. A CDC-supported depression workgroup, led by the UWHPRC, is conducting an evidence-based review of community interventions to prevent or alleviate depression. The nutrition interest group, under the leadership of Texas A&M University, is looking at the neighborhood food environment by examining the accessibility, affordability, and availability of healthy foods in communities where older adults live.

### Translation and dissemination

Led by the University of Colorado and the University of North Carolina at Chapel Hill, the research dissemination and practice workgroup has been the PRC–HAN's direct link to providing training to build state-based programs for healthy aging. The workgroup's goal is to help create local, state, and national community partnerships to build capacity and infrastructure in the public health and aging services networks and to provide more evidence-based health promotion opportunities for older adults. Key partners in this effort include the CDC, the Chronic Disease Directors, the Administration on Aging (AoA), the National Council on the Aging (NCOA), the National Association of State Units on Aging (NASUA), and the Agency for Healthcare Research and Quality (AHRQ). The workgroup plays a central role in developing materials to increase understanding of evidence-based interventions and disseminating these interventions and best practices.

### Capacity

To strengthen the network's capacity, the PRC–HAN is working to maximize relationships with partners and obtain new sources of funds for PRC–HAN priorities. Developing new partnerships has made it possible to enhance rather than duplicate efforts. For example, PRC–HAN members are supporting NCOA's development of a National Resource Center (www.healthyagingprograms.org) for AoA's evidence-based grants by developing and disseminating technical assistance topic papers and manuals for the AoA grantees. The PRC–HAN also collaborated with Medstat, the Centers for Medicare & Medicaid Services, NASUA, and NCOA to plan a demonstration of the Senior Risk Reduction Program, which will evaluate health risk appraisal systems in target communities nationwide. The PRC–HAN's contribution to planning the study was to develop a sampling frame that allows researchers to assess the usefulness of community support systems for behavior change. For example, ensuring that a known number of communities with adequate capacity (e.g., physical activity programs or information and assistance capability) are included in the sample would facilitate assessment. The network is also strengthening capacity by pooling its resources with those of its partners to generate new funding for PRC–HAN priorities. For example, the PRC–HAN successfully obtained funding from The Robert Wood Johnson Foundation for a *best practices* project in partnership with NCOA. Finally, fostering the development of workgroups enhances PRC–HAN functioning, strengthens the capacity of the individual members, and promotes synergy among network members. The depression workgroup, for example, has benefited from the contributions of researchers from both within and outside the PRC–HAN.

### Conference planning and communications

The PRC–HAN will stimulate the adoption of policies and programs by engaging policymakers, planners, and practitioners. The conference support workgroup, lead by UWHPRC, is designing a series of conferences to disseminate the lessons learned from our current work in physical activity and future work on depression and nutrition. The planning committee worked with two HAN-affiliated groups, the PRC–HAN community committees and representatives from the PRC–HAN research dissemination and practice workgroup, to ascertain their interests and needs for the design and content of the conferences. Presenters for the conferences include nationally known scholars in the field of public health, aging, and evidence-based programs in the areas of physical activity, depression, and nutrition. Such efforts are aimed at developing teams of public health and aging services practitioners, community members, and policymakers from states.

## Consequences

An evaluation of the PRC-HAN is important from several perspectives, including those of the CDC's Healthy Aging and PRC programs, PRC–HAN members, PRC–HAN community committees, other partners, and funders. In 2003, the PRC–HAN initiated an internal evaluation to better define the network's contributions to healthy aging, formalize internal processes, and better equip itself to serve as a model for other PRC thematic networks. The process began with internal interviews that led to a retreat to start developing a PRC–HAN logic model. The PRC–HAN is refining its logic model, developing performance indicators, and conducting a pilot evaluation to ensure that the PRC–HAN evaluation can be integrated into the PRC national evaluation.

## Interpretation

By making partnership, shared responsibility, and broad expertise key components of its capacity, the PRC–HAN has earned the recognition of other organizations as an important partner in advancing science-based health promotion activities for older adults. Its work in the area of physical activity for older adults brought additional partners to the network, which in turn has increased the diversity of the group's expertise and provided new outlets for its products. Such expansion also enables the network to increase its resources and engage in new project areas. Established trust and mutual respect among participants, strong ties to local communities, and the ability to combine its expertise in healthy aging with that of partners in national, state, and local organizations have positioned the PRC–HAN to continue as a productive and effective research network for years to come.

## Figures and Tables

**Table:** Research Agenda of the Prevention Research Centers Healthy Aging Research Network (PRC–HAN)

**Diseases, Organ System Topics, Geriatric Syndromes, and Concerns**	
Cardiovascular (coronary heart disease, hypertension, stroke) Arthritis and related conditions Osteoporosis Diabetes Cancer (colorectal, prostate, breast) Pulmonary disease Sensory impairments (vision, hearing) Depression and mental health Elder abuse and neglect	Chronic pain Frailty Falls, fractures, and other injuries Cognitive impairment or decline Incontinence Sleep disorders Oral health Polypharmacy Caregiving burden

**Health-Promoting Skills and Behaviors**	

Physical activity Nutrition (including undernutrition) Avoidance of tobacco use and substance abuse Self-management skills for personal behaviors, chronic disease management, and medication use Awareness and appropriate use of clinical preventive services Social engagement: participation in meaningful activity and social networks	

**Knowledge Domains — Research to Policy**	

Incidence and prevalence of risk factors Impact of risk factors on health and well-being Mechanisms associated with the adherence to changes (e.g., in behaviors, skills) Evidence that interventions for risk factor work to change behavior in older adult populations Policy and ecological strategies that could be used to incorporate change in risk factors or health in older adult populations	
